# Orbital T-Cell Lymphoma with Discrete Enlargements of All Extraocular Muscles Bilaterally in Patient with Moon Face Countenance

**DOI:** 10.1155/2017/8902162

**Published:** 2017-04-09

**Authors:** Hideaki Kawakami, Kiyofumi Mochizuki, Hideko Goto, Naoki Watanabe, Takuji Tanaka

**Affiliations:** ^1^Department of Ophthalmology, Gifu Municipal Hospital, Gifu, Japan; ^2^Department of Ophthalmology, Gifu University Graduate School of Medicine, Gifu, Japan; ^3^Department of Hematology, Gifu Municipal Hospital, Gifu, Japan; ^4^Department of Pathology, Gifu Municipal Hospital, Gifu, Japan

## Abstract

*Purpose*. To report our findings in a case of orbital T-cell lymphoma in which all of the extraocular muscles (EOMs) were bilaterally and discretely enlarged and the patient had a moon face countenance.* Case*. A 59-year-old woman presented with visual disturbances in her left eye, hyperemia in both eyes, and a moon face countenance. Examinations showed limited upward gaze in the right eye, blepharoptosis, hypertropia, and limited downward and rightward gaze in the left eye. Slit-lamp examination showed only chemosis and hyperemia of both eyes. Magnetic resonance imaging with contrast revealed discrete enlargements of the muscle bellies in all EOMs without abnormalities of the orbital fat in both eyes. Blood examinations excluded thyroid- and IgG4-related ophthalmopathy, and EOM biopsy revealed peripheral T-cell lymphoma. After beginning aggressive chemotherapy, the enlarged EOMs, limited eye motility, and moon face countenance improved. Unfortunately, the patient died of sepsis during the chemotherapy.* Conclusions*. A lymphoma should be included in the differential diagnosis of eyes with enlarged EOMs. Because lymphomas can lead to death, it is important for clinicians to consider lymphomas in eyes with enlarged EOMs.

## 1. Introduction

The incidence of lymphomas is increasing because of the growing number of older individuals, an increase in the number of patients with the acquired immunodeficiency syndrome, and improvements in diagnostic techniques. Orbital lymphomas make up 1.0 to 2.0% of all systemic lymphomas and most have a B-cell lineage [1]. T-cell lymphomas make up only 3 to 11% of the orbital lymphomas [1, 2]. Orbital lymphomas are mostly unilateral [2, 3], and the most common sites are the eyelid, conjunctiva, lacrimal gland, and orbital fat tissues. An involvement of the extraocular muscles (EOMs) is rare (0.17%) [1], and when present, it mostly invades a single EOM [1, 3].

We present our findings in a case of orbital peripheral T-cell lymphoma which was present in both eyes with discrete spindle-shaped enlargements of all of the extraocular muscles. In addition, the patient had a moon face countenance. Informed consent was obtained from the patient.

## 2. Case Report

**Figure 1:**
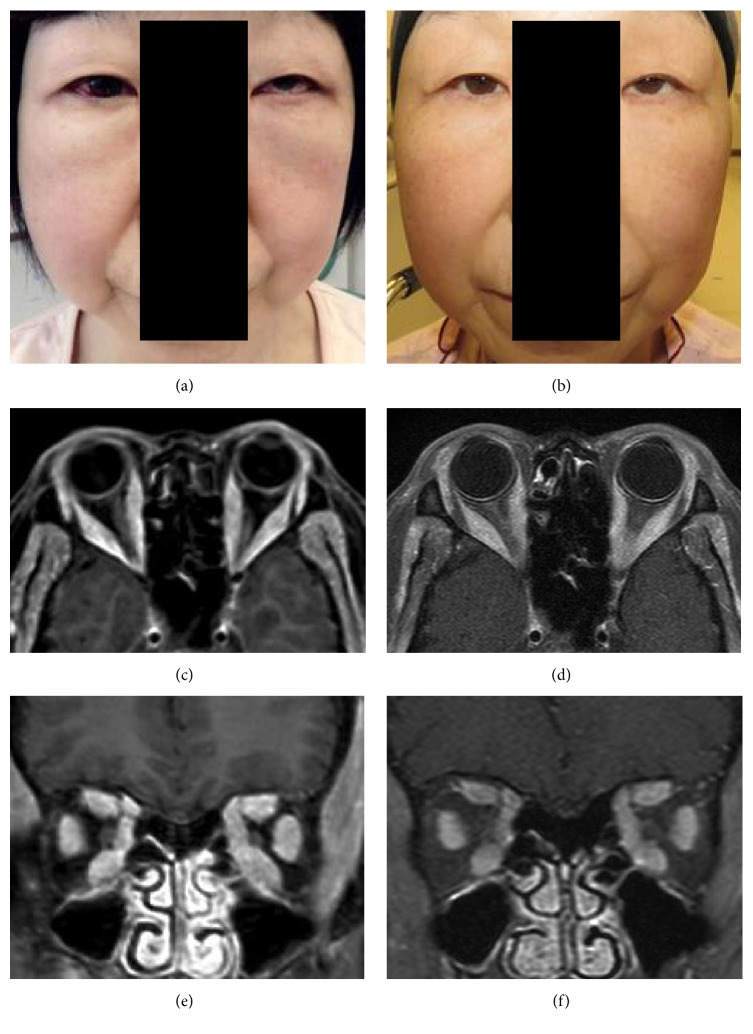
External facial photograph and enhanced magnetic resonance (MR) images with contrast of a patient diagnosed with orbital T-cell lymphoma. (a) External photograph shows moon face countenance at the initial examination. (b) External photograph shows relief of moon face countenance 1.5 months after aggressive chemotherapy with alternate hyper CVAD therapy (cyclophosphamide, vincristine, adriamycin, and dexamethasone), and MA therapy (methotrexate and cytarabine) was performed. (c, e) MR images showing an enlargement of all of the extraocular muscles at presentation. (d, f) MR images showing an attenuation of such findings at 1.5 months during the chemotherapy.

A 59-year-old woman presented with visual disturbances of her left eye and hyperemia of both eyes. She was generally in good health at presentation except for a moon face countenance without pain (Figure 1). She had noted a swelling of her face 1 year earlier, and she had seen an internal medicine doctor at general hospital. However, the general systemic examination had detected no abnormal findings in her body. Then she developed stiffness on the right side of her neck 3 months earlier and visited the Otolaryngology Department in our hospital. Echography and computed tomography revealed enlargements of the lacrimal glands bilaterally, the EOMs bilaterally, and the parotid gland on the right side. She had biopsies of the parotid gland on the right side twice, and histopathological examinations showed that the specimens were within normal limits. She visited our department because of abnormality of her eye position.

Our ophthalmic examination showed that her visual acuity was 20/25 OU, and Hertel exophthalmometer measurements were 15 mm OU. Blepharoptosis and hypertropia were found in her left eye. Motility examinations revealed a limited upward gaze in the right eye and limited downward and rightward gaze in the left eye. However, she did not have diplopia or eye pain. Slit-lamp examinations detected only the chemosis and hyperemia of both eyes. Her fundi were normal.

Magnetic resonance imaging with contrast showed discrete spindle-shaped enlargements of the bellies of all of the EOMs without a spillover into the orbital fat of both eyes (Figure 1). In addition, enlargements of the lacrimal and parotid glands and hyperplasia of the subcutaneous tissue in her face were detected in the MR images.

The results of peripheral blood examinations including WBC, CRP, serum IL-2, and IgG4, and thyroid-related hormones were within normal limits. However, the LDH and ferritin values were slightly higher than the standard level. Tests for adult T-cell leukemia and human immunodeficiency virus antibodies were negative, but those for Epstein-Barr virus were positive. Systemic examinations for lymphoma by gallium scintigraphy, flow cytometry, bone marrow aspirate and trephine, and lumbar puncture detected no abnormal findings.

**Figure 2:**
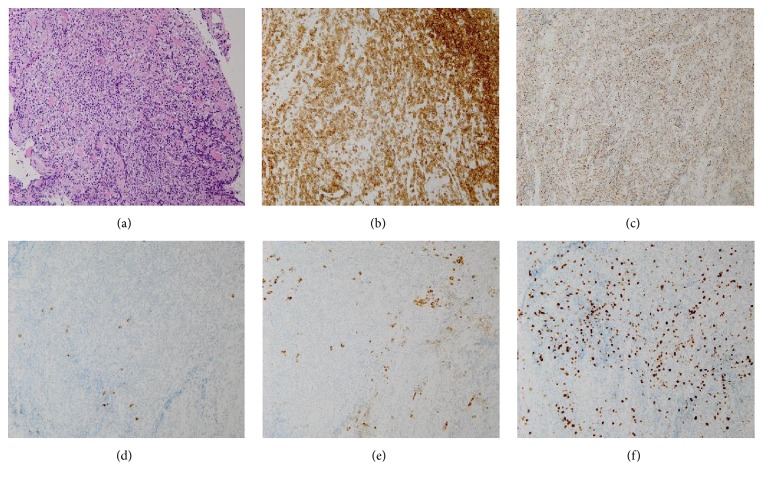
Photomicrographs of the inferior rectus muscle with peripheral T-cell lymphoma. (a) Photomicrograph showing infiltration by many lymphocytes into the muscles (hematoxylin and eosin stain; magnification, ×200). (b)–(f) Photomicrographs showing positivity for CD3 (b) and Granzyme B (c) and negativity for CD20 (d), CD79a (e), and MIB-1 index in 60% (f) (immunohistochemistry stain; magnification, ×200).

Biopsy was performed, and histopathological examinations of the biopsies of the inferior rectus muscle of her left eye and the parotid gland on the right side showed an infiltration of atypical cells into the tissues. Immunohistochemical stains showed that the cells were positive for CD3, CD5, CD8, and Granzyme B (T-cell markers) and negative for CD20, CD56, and CD79a (B-cell markers) (Figure 2). Based on these results, she was diagnosed with stage 4, peripheral T-cell lymphoma.

Aggressive chemotherapy for T-cell lymphoma was performed with alternate hyper CVAD therapy (cyclophosphamide, vincristine, adriamycin, and dexamethasone) and MA therapy (methotrexate and cytarabine). After the initiation of chemotherapy, her enlarged EOMs, limited eye motility, and moon face countenance were improved (Figure 2). Unfortunately, she died of sepsis 8 weeks after the initiation of the chemotherapy.

## 3. Discussion

Enlarged EOMs are known to be associated with thyroid- and IgG4-related diseases, orbital myositis, carotid cavernous fistulas, and metastatic tumors (Table 1) [4]. The incidence of enlarged EOMs in thyroid ophthalmopathy is from 80 to 90% [4], and that for IgG4-related ophthalmic disease is 10 to 89% [5, 6]. The incidence of enlarged EOMs is considerably higher for these diseases. Lymphomas can also cause an enlargement of the EOMs although the incidence is low at 0.17 to 13% [1, 7]. In addition, the number of infiltrated EOMs in eyes with an orbital lymphoma is usually one in 82 to 100% of the cases [1, 3]. The rate of unilateral involvement was 90 to 100% [1–3]. Both thyroid- and IgG4-related diseases tend to have several enlarged EOMs, and thyroid orbitopathy is bilateral in 70% of the cases and IgG4-related disease in 70 to 88% of the cases (Table 1) [4, 5].

Most of orbital lymphomas can have a spillover into the orbital fat as seen in MR images. The MR images show that the lymphoid infiltration causes muscle swelling with indistinct borders and can involve the tendons. The swelling is more massive than in myositis [1, 3, 8]. A spindle-shaped enlargement of the EOM is often present in thyroid ophthalmopathy, IgG4-related ophthalmic myositis, and carotid cavernous fistulas (Table 1) but rarely in lymphomas. In addition, an involvement of the EOMs in orbital lymphomas tends to occur in the superior rectus muscle and levator muscle (Table 1) [3–5]. The spindle-shape involvement of the all EOMs without spillover into the orbital fat in the MR images as in our case is quite rare in orbital lymphomas.

A moon face countenance results from long-term use of steroid medications and benign tumors or cancers of the adrenal gland, lung, pancreas, thymus, and pituitary gland. In addition, both thyroid- and IgG4-related diseases can also be associated with a moon face countenance. Our case had none of these conditions, and no disorders were detected by systemic examination. In addition, the moon face countenance was improved by chemotherapy (Figure 1). Therefore, we assume that the cause of the moon face countenance would be due to infiltration of lymphocytes into the facial tissues.

Therefore, it is difficult for clinicians to initially consider an orbital lymphoma in a patient with bilateral and discrete, spindle-shaped enlargements of the EOMs with a moon face countenance as in our case. Such a case can easily be misdiagnosed as thyroid ophthalmopathy or IgG4-related ophthalmic disease [1, 5, 9].

Peripheral T-cell lymphomas tend to develop in 55- to 60-year-old men of East Asian ancestry. The predisposing factors for T-cell lymphomas are gene translocation, Epstein-Barr virus, noxious chemicals, and smoking, but the exact mechanism has not been determined. Our case was 59-year-old Japanese woman whose only sign was being positive for the Epstein-Barr virus antibody.

T-cell lymphomas have a more aggressive course [1], and most of the patients often initially visit at stage 3 or 4. There is no standard therapy for T-cell lymphomas, therefore the patients with T-cell lymphoma are usually treated with chemotherapy as well as those with B-cell lymphomas. However, T-cell lymphomas are treatment-resistant and have a greater rate of recurrences than B-cell lymphomas [1]. The prognosis is poor with a 5-year survival rate of 20 to 30% [10]. Bilateral orbital lymphomas have 3.89 times higher mortality than unilateral orbital lymphomas with a mortality rate of 40% [1, 7]. Our case had a definitive diagnosis made more than 1 year after the appearance of the signs and symptoms and 3 months after the initial examination. Her condition had been the peripheral T-cell lymphoma of stage 4 and bilateral in orbit at presentation. Consequently, she died 8 weeks after the initiation of chemotherapy, although the cause of death was sepsis.

A misdiagnosis or diagnostic delay of lymphomas can be fatal. Therefore, repeated histopathological examinations of biopsies with more detailed clinical findings are necessary for a definitive diagnosis so that appropriate therapy can be initiated. We recommend that clinicians consider lymphomas in the differential diagnosis of enlarged EOMs even if the enlargement of the EOMs is spindle-shaped and occurs bilaterally and discretely.

## Figures and Tables

**Table 1:** Characteristics of diseases causing an enlargement of the extraocular muscles.

	Lymphoma	Thyroid ophthalmopathy	IgG4-related ophthalmic myositis	Idiopathic orbital myositis	Carotid cavernous fistula	Metastatic tumor
Age	60	40–60	40–60	30–40	60–70	50–60
Male to female	1 : 1	1 : 5	1 : 1	1 : 2	1 : 3	Male ≧ Female
Incidence of enlarged EOM (%)	0.17–13	80–90	10–50	100	65	9
Bilateral involvements of enlarged EOM (%)	≦10	70	70–90	40–50	Few	10–20
Number of affected EOM	One (82–100%)	Multiple	Multiple	One (66%)	Multiple	One (91%)
Common involved EOM	SRM > IRM	IRM > MRM > SRM	LRM > IRM	Almost equal numbers in 4 RMs	MRM = LRM > SRM	MRM > LRM
Shape of EOM involved	Spindle-like enlargement	Spindle-like enlargement	Spindle-like enlargement	Tendon involvement (stick-like enlargement)	Spindle-like enlargement	NA
Onset form	Chronic	Chronic	Chronic	Acute	Acute	Chronic
Clinical findings	Proptosis, diplopia, blepharoptosis	Proptosis, diplopia, Graefe's sign, Dalrymple's sign, and Stellwag's sign	Proptosis, diplopia, and enlargement of lacrimal gland and trigeminal nerve	Proptosis, diplopia, eye pain, chemosis, hyperemia, lid swelling, and blepharoptosis	Conjunctival cork screw vessels, proptosis, double vision, bruit, pulsating exophthalmos, dilatation and tortuosity of superior ophthalmic vein and retinal central vein, and elevated IOP	Proptosis, diplopia, blepharoptosis, and eye pain

EOM: extraocular muscle, SRM: superior rectus muscle, IRM: inferior rectus muscle, MRM: medial rectus muscle, LRM: lateral rectus muscle, IOP: intraocular pressure, and NA: not available data.
